# Targeted temperature management guided by the severity of hyperlactatemia for out-of-hospital cardiac arrest patients: a post hoc analysis of a nationwide, multicenter prospective registry

**DOI:** 10.1186/s13613-019-0603-y

**Published:** 2019-11-19

**Authors:** Tomoya Okazaki, Toru Hifumi, Kenya Kawakita, Yasuhiro Kuroda, the Japanese Association for Acute Medicine out-of-hospital cardiac arrest (JAAM-OHCA) registry

**Affiliations:** 1 Emergency Medical Center, Kagawa University Hospital, 1750-1 Ikenobe, Kita, Miki, Kagawa 761-0793 Japan; 2 Department of Emergency and Critical Care Medicine, St. Luke’s International Hospital, 9-1 Akashi-cho, Chuo-ku, Tokyo, 104-8560 Japan

**Keywords:** Out-of-hospital cardiac arrest, Hyperlactatemia, Targeted temperature management

## Abstract

**Background:**

The International Liaison Committee on Resuscitation guidelines recommend target temperature management (TTM) between 32 and 36 °C for patients after out-of-hospital cardiac arrest, but did not indicate patient-specific temperatures. The association of serum lactate concentration and neurological outcome in out-of-hospital cardiac arrest patient has been reported. The study aim was to investigate the benefit of 32–34 °C in patients with various degrees of hyperlactatemia compared to 35–36 °C.

**Methods:**

This study was a post hoc analysis of the Japanese Association for Acute Medicine out-of-hospital cardiac arrest registry between June 2014 and December 2015. Patients with complete targeted temperature management and lactate data were eligible. Patients were stratified to mild (< 7 mmol/l), moderate (< 12 mmol/l), or severe (≥ 12 mmol/l) hyperlactatemia group based on lactate concentration after return of spontaneous circulation. They were subdivided into 32–34 °C or 35–36 °C groups. The primary endpoint was an adjusted predicted probability of 30-day favorable neurological outcome, defined as a cerebral performance category score of 1 or 2.

**Result:**

Of 435 patients, 139 had mild, 182 had moderate, and 114 had severe hyperlactatemia. One hundred and eight (78%) with mild, 128 with moderate (70%), and 83 with severe hyperlactatemia (73%) received TTM at 32–34 °C. The adjusted predicted probability of a 30-day favorable neurological outcome following severe hyperlactatemia was significantly greater with 32–34 °C (27.4%, 95% confidence interval: 22.0–32.8%) than 35–36 °C (12.4%, 95% CI 3.5–21.2%; *p* = 0.005). The differences in outcomes in those with mild and moderate hyperlactatemia were not significant.

**Conclusions:**

In OHCA patients with severe hyperlactatemia, the adjusted predicted probability of 30-day favorable neurological outcome was greater with TTM at 32–34 °C than with TTM at 35–36 °C. Further evaluation is needed to determine whether TTM at 32–34 °C can improve neurological outcomes in patients with severe hyperlactatemia after out-of-hospital cardiac arrest.

## Background

Out-of-hospital cardiac arrest (OHCA) is associated with high mortality and poor neurological outcomes [1]. Targeted temperature management (TTM) is often used to treat comatose OHCA patients after the return of spontaneous circulation (ROSC) [2–4]. The International Liaison Committee on Resuscitation guidelines strongly recommend TTM between 32 and 36 °C [5, 6] but did not provide patient-specific target temperatures. Precision medicine allows mild therapeutic hypothermia or normothermia to be chosen for individual patients, based on the initial brain damage [7]. Specific OHCA patient characteristics that can predict the benefits of 32–34 °C compared with 35–36 °C [8–12] have not been identified.

Serum lactate concentration is a nonspecific marker of tissue hypoxia, aerobic glycolysis, and lactate clearance [13]. The association of serum lactate and neurological outcome has been described in OHCA [14–17] but the differential benefits of TTM at 32–34 °C and TTM at 35–36 °C in patients with different initial serum lactate concentrations have not been reported.

We hypothesized that the effects of TTM at 32–34 °C had different influence according to the severity of hyperlactatemia after ROSC. The study aim was to investigate the benefit of TTM at 32–34 °C in OHCA patients with various degrees of hyperlactatemia compared to TTM at 35–36 °C.

## Methods

### Study design, setting, and patient selection

This study was a post hoc analysis of patients who were included in the Japanese Association for Acute Medicine out-of-hospital cardiac arrest (JAAM-OHCA) registry between June 2014 and December 2015. The registry is a nationwide, multicenter prospective registry that included 56 institutions in 2014 and 73 in 2015. The JAAM-OHCA registry collected both pre- and post-hospitalization data. Prehospitalization data were obtained from the All Japan Utstein Registry of the Fire and Disaster Management Agency as previously reported [18]. In-hospital data were collected via an Internet-based system by physicians or medical staff at each institution. The JAAM-OHCA registry committee integrated the prehospital and in-hospital data, as previously described [1]. The protocol was approved by the Institutional Review Board of each participating hospital. Comatose adult OHCA patients with TTM treatment after ROSC were included and patients with withdrawn TTM were excluded.

### Data collection

Patient age, sex, witness status, presence of dispatcher instruction, presence of a bystander who performed cardiopulmonary resuscitation (CPR), etiology of cardiac arrest (cardiac or not), initial cardiac rhythm, prehospital epinephrine administration, prehospital airway management, prehospital automated external defibrillator (AED) use, response time (time from call to contact with a patient), time from call to hospital arrival, prehospital ROSC, Glasgow Coma Scale score, coronary angiography, use of a mechanical circulatory device (extracorporeal membrane oxygen and/or intra-aortic balloon pumping), door-to-first ABG measurement after ROSC, arterial blood gas measurement data including serum lactate concentration after ROSC, door-to-TTM initiation, target temperature during TTM, and neurological outcome 30 days after cardiac arrest were included in the analysis. The responsibility for setting target temperature was entrusted entirely to each physician or each institution. There were no specific protocols of TTM for the JAAM-OHCA registry.

### Primary exposure

Patients were divided by serum lactate concentration after ROSC into mild (< 7 mmol/l), moderate (< 12 mmol/l), or severe (≥ 12 mmol/l) hyperlactatemia group. The cutoff values were chosen for reasons that were previously described [15, 16]. Then, patients in each hyperlactatemia group were subdivided into 32–34 °C and 35–36 °C groups based on their target temperature.

### Outcome measures

The primary endpoint was an adjusted predicted probability of 30-day favorable neurological outcome. A favorable neurological outcome was defined as a cerebral performance category (CPC) score of 1 or 2 [19]. The CPC score includes five outcomes, including 1, good cerebral recovery; 2, moderate cerebral disability; 3, severe cerebral disability; 4, coma or vegetative state; and 5, death/brain death [20]. The secondary outcome was an adjusted predicted probability of 30-day survival.

### Statistical analysis

To obtain the adjusted predicted probabilities of 30-day favorable neurological outcome and 30-day survival, we used multiple logistic regression model adjusted for age > 65 [21, 22], gender [21, 23], witness [24], dispatcher instruction [25, 26], bystander CPR [24, 27], cardiac etiology [28], initial shockable rhythm [29, 30], prehospital epinephrine administration [31], prehospital advanced airway management [32], time from call to hospital arrival [33, 34], prehospital ROSC [35, 36], Glasgow Outcome Scale score [37], coronary angiography [38], extracorporeal membrane oxygenation (ECMO) and/or intra-aortic balloon pumping (IABP) [22, 39], and PaCO_2_ 30–50 mm Hg [40, 41], as performed in previous studies [23, 42].

We firstly examined the comparison between patients finally analyzed and those excluded. Then, we assessed the association of serum lactate after ROSC and both crude favorable neurological outcomes and the adjusted predicted probability of favorable neurological outcomes in the patients finally analyzed. Furthermore, study participants were divided into 32–34 °C and 35–36 °C groups in each hyperlactatemia category based on primary exposure. We compared the adjusted predicted probabilities of favorable neurological outcomes and survival between 32–34 and 35–36 °C groups in each hyperlactatemia category. We calculated *p* value for interaction between hyperlactatemia group and TTM (32–34 °C or 35–36 °C) for the adjusted predicted probabilities of 30-day favorable neurological outcome and 30-day survival.

Statistical analysis was performed with EZR software (Saitama Medical Center, Jichi Medical University, Saitama, Japan) [43]. Continuous variables were compared with Mann–Whitney *U* or Kruskal–Wallis tests. Binary variables were compared with Fisher’s exact test. *p* values < 0.05 were considered statistically significant. Missing data were not replaced or estimated.

## Results

### Baseline patient characteristics

**Fig. 1:**
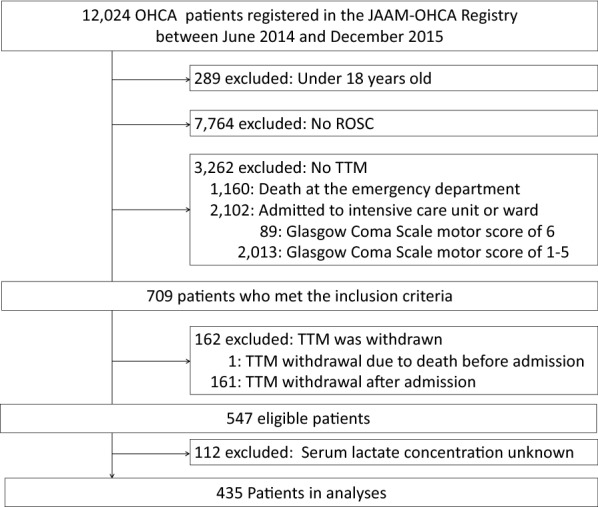
Flow diagram of study

A total of 12,024 OHCA patients were included in the OHCA registry during the study period. Of 3971 adult patients with ROSC, there were 3262 patients without TTM treatment, 162 with withdrawn TTM, and 112 without lactate concentration after ROSC. Then, 435 were included in the final analysis (Fig. 1). Favorable neurological outcomes were observed in 41.8% of the study patients. Baseline characteristics between the patients excluded and those included are shown in Table 1.

**Table 1:** Patient baseline characteristics between inclusion group and three exclusion groups

Variables	Inclusion *n* = 435	No TTM *n* = 2013^a^	*p* value^c^	TTM withdrawal *n* = 161^b^	*p* value^c^	Unknown lactate concentration *n* = 112	*p* value^c^
Age, years	63 [50, 73]	75 [6, 83]	< 0.001	65 [53, 75]	0.126	63 [51, 72]	0.881
Age > 65 years	182 (42)	1405 (70)	< 0.001	78 (48)	0.163	51 (46)	0.521
Male sex	338 (78)	1190 (59)	< 0.001	115 (71)	0.130	85 (76)	0.705
Witness	348 (80)	1312 (65)	< 0.001	122 (76)	0.261	83 (74)	0.195
Dispatcher instruction	198 (46)	852 (42)	0.24	75 (47)	0.853	45 (40)	0.338
Bystander-performed CPR	228 (52)	839 (42)	< 0.001	88 (55)	0.645	50 (45)	0.168
Cardiac etiology	332 (76)	786 (39)	< 0.001	128 (80)	0.443	85 (76)	0.902
Initial shockable rhythm	235 (54.0)	218 (11)	< 0.001	74 (46)	0.096	54 (48)	0.290
Prehospital epinephrine administration	99 (23)	613 (31)	0.001	57 (35)	0.001	29 (26)	0.532
Prehospital advanced airway management	145 (33)	871 (43)	< 0.001	61 (38)	0.332	43 (38)	0.318
Prehospital AED	313 (72)	777 (39)	< 0.001	110 (68)	0.416	73 (65)	0.165
Response time, min	8 [6, 9]	8 [7, 10]	< 0.001	8[7, 10]	< 0.001	8 [6, 10]	0.197
Time from call to hospital arrival, min	30 [24, 36]	32 [27, 40]	< 0.001	32 [27, 40]	0.014	30 [24, 36]	0.942
Prehospital ROSC	252 (58)	563 (28)	< 0.001	36 (22)	< 0.001	28 (25)	< 0.001
Glasgow Coma Scale score	3 [3, 3]	3 [3, 3.]	< 0.001	3 [3, 3]	< 0.001	3 [3, 3]	0.003
Coronary angiography	257 (59)	235 (12)	< 0.001	100 (62)	0.512	75 (67)	0.131
ECMO and/or IABP	123 (28)	157 (8)	< 0.001	76 (47)	< 0.001	54 (48)	< 0.001
Targeted temperature management			NA		0.198		0.005
32–34 °C	319 (73)	0 (0)		124 (79)		92 (86)	
35–36 °C	116 (27)	0 (0)		33 (21)		15 (14)	
30-day favorable neurological outcome	182 (42)	183 (9)	< 0.001	20 (12)	< 0.001	32 (29)	0.012
30-day survival	325 (75)	354 (18)	< 0.001	36 (22)	< 0.001	67 (60)	0.003

Continuous variables were presented as median [Interquartile range]. Categorical variables were presented as *n* (%)

*TTM* targeted temperature management, *CPR* cardiopulmonary resuscitation, *AED* automated external defibrillator, *ROSC* return of spontaneous circulation, *ECMO* extracorporeal membrane oxygenation, *IABP* intra-aortic balloon pumping, *ABG* arterial blood gas

^a^There were 1160 patients excluded due to death at the emergency department and 89 patients excluded because they got Glasgow Coma Scale motor score of 6 after return of spontaneous circulation

^b^There was one patient excluded due to death at the emergency department

^c^Vs. inclusion group

### Association of serum lactate concentrations after ROSC and favorable neurological outcomes

The crude and adjusted predicted probability of a favorable 30-day neurological outcome across the range of serum lactate concentrations after ROSC is shown in Additional file 1: Figure S1. We found a trend toward decreases of both crude and adjusted predicted probability of a favorable 30-day neurological outcome with increasing serum lactate concentration. There were 139 patients in the mild, 182 in the moderate, and 114 in the severe hyperlactatemia groups. Table 2 shows the characteristics of the patients in each hyperlactatemia group. There were significant differences among the three groups in witness, initial shockable rhythm, prehospital epinephrine administration, prehospital advanced airway management, prehospital AED, prehospital ROSC, Glasgow coma scale score, ECMO and/or IABP, door-to-first arterial blood gas measurement after ROSC, pH, PaCO_2_, HCO_3_, and base excess. The percentage of patients with favorable neurological outcomes decreased with the worsening of hyperlactatemia: 60.4% with mild, 41.8% with moderate, and 19.3% with severe hyperlactatemia had favorable neurological outcomes.

**Table 2:** Comparison between three hyperlactatemia groups

Variables	Hyperlactatemia classification			*p* value
	Mild *n* = 139	Moderate *n* = 182	Severe *n* = 114	
Age, years	64 [52, 73]	64 [50, 75]	60 [48, 68]	0.087
Age > 65 years	63 (45)	81 (45)	38 (33)	0.098
Male sex	105 (76)	143 (79)	90 (79)	0.757
Witness	118 (85)	149 (82)	81 (71)	0.017
Dispatcher instruction	66 (47)	89 (49)	43 (38)	0.149
Bystander-performed CPR	77 (55)	100 (55)	51 (45)	0.161
Cardiac etiology	115 (83)	135 (74)	82 (72)	0.089
Initial shockable rhythm	88 (63)	100 (55)	47 (41)	0.002
Prehospital epinephrine administration	21 (15)	41 (23)	37 (32)	0.005
Prehospital advanced airway management	33 (24)	61 (34)	51 (45)	0.002
Prehospital AED	114 (82)	132 (73)	67 (59)	< 0.001
Response time, min	8 [6, 9]	8 [6, 9]	8 [7, 9]	0.376
Time from call to hospital arrival, min	32 [25, 39]	30 [24, 35]	29 [24, 37]	0.200
Prehospital ROSC	102 (73)	112 (62)	38 (33)	< 0.001
Glasgow Coma Scale score	3 [3, 4]	3 [3, 3]	3 [3, 3]	< 0.001
Coronary angiography	85 (61)	106 (58)	66 (58)	0.833
ECMO and/or IABP	45 (32)	37 (20)	41 (36)	0.006
Door-to-first ABG measurement after ROSC, min	11 [5, 30]	9 [5, 18]	15 [7, 30]	0.007
pH	7.28 [7.22, 7.33]	7.12 [7.02, 7.28]	6.92 [6.75, 7.03]	< 0.001
PaCO_2_, mm Hg	43 [37, 51]	52 [35, 75]	70 [43, 97]	< 0.001
PaCO_2_ 30–50 mm Hg	90 (65)	63 (35)	27 (24)	< 0.001
PaO_2_, mm Hg	176 [91, 337]	187 [92, 356]	150 [79, 346]	0.760
HCO_3_, mmol/l	19.9 [17.1, 21.7]	16.4 [13.9, 18.6]	12.9 [10.3, 16.8]	< 0.001
Base excess, mmol/l	− 6.2 [− 9.1, − 4.0]	− 12 [− 14.7, − 9.5]	− 19.4 [− 24.1, − 15.4]	< 0.001
Lactate value, mmol/l	5.2 [3.7, 6.1]	9.1 [7.9, 10.2]	14.9 [13.2, 18.0]	< 0.001
Door-to-TTM initiation, min	106 [30, 188]	115 [36, 190]	136 [46, 225]	0.324
Targeted temperature management				0.331
32–34 °C	108 (78)	128 (70)	83 (73)	
35–36 °C	31 (22)	54 (30)	31 (27)	
30-day favorable neurological outcome	84 (60)	76 (42)	22 (19)	< 0.001
30-day survival	117 (84)	144 (79)	64 (56)	< 0.001

Continuous variables were presented as median [Interquartile range]. Categorical variables were presented as *n* (%)

*CPR* cardiopulmonary resuscitation, *AED* automated external defibrillator, *ROSC* return of spontaneous circulation, *ECMO* extracorporeal membrane oxygenation, *IABP* intra-aortic balloon pumping, *ABG* arterial blood gas

### Baseline characteristics of patients stratified by hyperlactatemia and treated with 32–34 °C or 35–36 °C

Comparison of the baseline characteristics of patients with mild, moderate, or severe hyperlactatemia and treated with either 32–34 °C or 35–36 °C are summarized in Table 3. The analysis revealed significant differences in initial shockable rhythm rates and door-to-TTM initiation time among the mild hyperlactatemia group, in ages and door-to-TTM initiation time among the moderate hyperlactatemia group, and door-to-TTM initiation time among the severe hyperlactatemia group.

**Table 3:** Comparison between 32–34 and 35–36 °C in each hyperlactatemia group

Variables	Mild hyperlactatemia		*p* value	Moderate hyperlactatemia		*p* value	Severe hyperlactatemia		*p* value
	32–34 °C *n* = 108	35–36 °C *n* = 31		32–34 °C *n* = 128	35–36 °C *n* = 54		32–34 °C *n* = 83	35–36 °C *n* = 31	
Age, years	64 [52, 72]	66 [54, 79]	0.105	62 [50, 74]	71 [51, 80]	0.036	58 [47, 67]	64 [55, 71]	0.112
Age > 65 years	47 (43.5)	16 (51.6)	0.540	50 (39)	31 (57)	0.033	24 (28.9)	14 (45.2)	0.121
Male sex	80 (74)	25 (81)	0.636	101 (79)	42 (78)	0.846	67 (81)	23 (74)	0.449
Witness	92 (85)	26 (84)	0.784	106 (83)	43 (80)	0.675	61 (74)	20 (65)	0.361
Dispatcher instruction	48 (44)	18 (58)	0.222	63 (49)	26 (48)	1.000	31 (37)	12 (39)	1.000
Bystander-performed CPR	58 (54)	19 (61)	0.540	73 (57)	27 (50)	0.417	39 (47)	12 (39)	0.527
Cardiac etiology	90 (83)	25 (81)	0.788	98 (77)	37 (69)	0.271	61 (74)	21 (68)	0.640
Initial shockable rhythm	74 (69)	14 (45)	0.021	75 (59)	25 (46)	0.144	36 (43)	11 (36)	0.524
Prehospital epinephrine administration	18 (17)	3 (10)	0.409	31 (24)	10 (19)	0.444	27 (33)	10 (32)	1.000
Prehospital advanced airway management	24 (22)	9 (29)	0.475	41 (32)	20 (37)	0.606	37 (45)	14 (45)	1.000
Prehospital AED	90 (83)	24 (77)	0.437	93 (73)	39 (72)	1.000	50 (60)	17 (55)	0.671
Response time, min	8 [6, 9]	8 [6, 10]	0.749	7 [6, 9]	8 [6, 9]	0.381	8 [6, 9]	8 [7, 11]	0.329
Time from call to hospital arrival, min	32 [25, 40]	31 [23, 33]	0.283	30 [24, 34]	31 [24, 36]	0.542	29 [25, 37]	28 [24, 39]	0.769
Prehospital ROSC	77 (71)	25 (81)	0.362	78 (61)	34 (63)	0.868	31 (37)	7 (23)	0.181
Glasgow Coma Scale score	3 [3, 3]	3 [3, 6]	0.097	3 [3, 3]	3 [3, 3]	0.602	3 [3, 3]	3 [3, 3]	0.856
Coronary angiography	66 (61)	19 (61)	1.000	78 (61)	28 (52)	0.324	52 (63)	14 (45)	0.135
ECMO and/or IABP	37 (34.3)	8 (25.8)	0.514	50 (39)	13 (24)	0.061	32 (39)	9 (29)	0.388
Door-to-first ABG measurement after ROSC, min	11 [5, 32]	9 [5, 22]	0.300	10 [5, 17]	9 [6, 18]	0.784	13 [6, 30]	19 [11, 35]	0.056
pH	7.28 [7.22, 7.32]	7.31 [7.20, 7.37]	0.107	7.14 [7.02, 7.28]	7.08 [7.01, 7.25]	0.356	6.92 [6.81, 7.03]	6.91 [6.74, 7.15]	0.817
PaCO_2_, mm Hg	43 [38, 51]	40 [33, 51]	0.318	47 [34, 75]	60 [36, 75]	0.200	69 [44, 90]	72 [31, 108]	0.873
PaCO_2_ 30–50 mm Hg	71 (65.7)	19 (61.3)	0.674	50 (39)	13 (24)	0.061	23 (28)	4 (13)	0.137
PaO_2_, mm Hg	170 [91, 350]	176 [93, 325]	0.975	179 [86, 354]	195 [113, 350]	0.449	148 [76, 345]	240 [86, 363]	0.437
HCO_3_, mmol/l	20.0 [16.8, 21.5]	19.9 [18.0, 22.8]	0.608	16.3 [13.9, 18.5]	16.6 [13.6, 19.5]	0.471	13.0 [10.4, 17.1]	12.7 [10.3, 15.1]	0.530
Base excess, mmol/l	− 6.4 [− 9.3, − 4.0]	− 5.7 [− 7.3, − 4.1]	0.369	− 11.9 [− 14.9, − 9.4]	− 12.5 [− 14.0, − 9.9]	0.905	− 19.6 [− 23.7, − 16.2]	− 19.4 [− 24.5, − 12.6]	0.703
Lactate value, mmol/l	5.1 [3.6, 6, 1]	5.4 [3.9, 6.5]	0.185	9.1 [7.9, 10.1]	9.3 [8.1, 10.6]	0.775	14.7 [13.1, 17.3]	16.2 [14.0, 19.4]	0.113
Door-to-TTM initiation, min	82 [20, 174]	167 [109, 213]	0.003	78 [21, 170]	164 [103., 218]	< 0.001	105 [35, 202]	198 [109, 261]	0.002
30-day favorable neurological outcome	65 (60)	19 (61)	1.000	56 (44)	20 (37)	0.417	18 (22)	4 (13)	0.425
30-day survival	89 (82)	28 (90)	0.406	100 (78)	44 (82)	0.693	48 (58)	16 (52)	0.672

Continuous variables were presented as median [Interquartile range]. Categorical variables were presented as *n* (%)

*CPR* cardiopulmonary resuscitation, *AED* automated external defibrillator, *ROSC* return of spontaneous circulation, *ECMO* extracorporeal membrane oxygenation, *IABP* intra-aortic balloon pumping, *ABG* arterial blood gas

### Favorable neurological outcomes and survival following 32–34 °C or 35–36 °C in the three hyperlactatemia groups

**Fig. 2:**
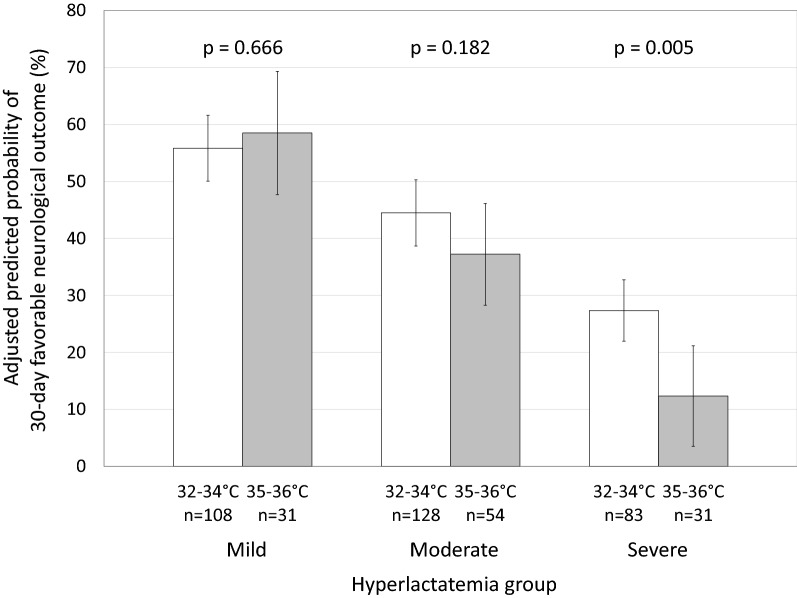
Adjusted predicted probability of favorable 30-day neurological outcomes of 32–34 °C and 35–36 °C among patients in the three hyperlactatemia group. Error bars indicate 95% confidence intervals. *p* for interaction = 0.043

Multiple logistic regression models to obtain the adjusted predicted probabilities of 30-day favorable neurological outcome showed that age older than 65 years, initial shockable rhythm, prehospital epinephrine administration, prehospital advanced airway management, time from call to hospital arrival, prehospital ROSC, Glasgow Coma Scale score, and PaCO_2_ 30–50 mm Hg were significantly associated with neurological outcome (Additional file 2: Table S1). There were no significant differences in the rates of crude and adjusted predicted 30-day favorable neurological outcomes following 32–34 °C and 35–36 °C in patients with mild or moderate hyperlactatemia. In patients with severe hyperlactatemia, the adjusted predicted probability of a favorable outcome, 27.4% (95% confidence interval (CI) 22.0–32.8%) with 32–34 °C and 12.4% (95% CI 3.5–21.2%) with 35–36 °C, were significantly different (*p* = 0.005, Fig. 2). The interaction between hyperlactatemia groups and TTM groups was significant (*p* for interaction = 0.043). Thirty-day survival decreased with the worsening of hyperlactatemia, from 84.2% with mild to 79.1% with moderate and 56.1% with severe hyperlactatemia (Table 2). Multiple logistic regression models to obtain the adjusted predicted probabilities of 30-day survival showed that initial shockable rhythm, prehospital epinephrine administration, time from call to hospital arrival, prehospital ROSC, and Glasgow Coma Scale score were significantly associated with 30-day mortality (Additional file 3: Table S2). Within each hyperlactatemia group, crude 30-day survival and adjusted predicted probability of 30-day survival with 32–34 °C or with 35–36 °C did not differ significantly (Table 3, Additional file 4: Figure S2).

## Discussion

This post hoc analysis of a nationwide, multicenter prospective registry found no significant differences in the adjusted predicted probability of favorable neurological outcomes with TTM at 32–34 °C or 35–36 °C in OHCA patients with mild (< 7 mmol/l) or moderate (< 12 mmol/l) hyperlactatemia after ROSC. TTM at 32–34 °C was associated with a greater adjusted predicted probability of a favorable outcome in patients with severe hyperlactatemia than TTM at 35–36 °C.

Two previous retrospective observational studies also reported that TTM at 32–34 °C was beneficial in patients with severe OHCA patients [8, 44]. One was a single-center cohort study including 1200 witnessed OHCA patients [8]. It was found that TTM at 32–34 °C was associated with improved neurological outcomes in patients with relatively longer times from collapse to the start of resuscitation attempts. The second study evaluated 871 witnessed, shockable adult patients prospectively included in a population-based registry in Toronto, Canada [44]. The beneficial effect of TTM at 32–34 °C was more apparent in patients with a prolonged time from collapse to initial defibrillation.

Two retrospective studies reported that lower target temperature improved outcome in OHCA patients with less severe baseline characteristics [11, 12]. One evaluated the outcomes in a cohort of Japanese patients treated with either low (32.0–33.5 °C) or moderate (34.0–35 °C) target temperatures and stratified to a short (< 30 min) or long interval to resuscitation [11]. Improved neurological outcomes were observed only in the short-interval group. However, a target temperature of 34 °C was classified as moderate target temperature group in this study, and more than 75% of patients in the moderate target temperature group had a target temperature of 34 °C. In case of classifying target temperature of 34 °C into low temperature group as in our current study or other studies [8, 12, 44], patient characteristics and main results may be subject to a major change. Thus, it is very difficult to compare this study and our current study at present. Another retrospective observational study included patients with or without early evidence of hypoxic encephalopathy provided by brain computed tomography [12]. Compared to TTM at 35–36 °C, the benefit of TTM at 34 °C was evident only in patients without hypoxic encephalopathy. In that study, the brain damage resulting from hypoxic encephalopathy was considered to be too severe to permit a favorable response to TTM. The limited available data suggest that patients with severe OHCA benefit from TTM at 32–34 °C, as shown in this study, but the effect may not be seen in extremely severe cases.

Serum lactate concentrations reflect not only tissue hypoxia but also aerobic glycolysis and lactate clearance in critically ill patients [13]. Several observational studies have shown that serum lactate concentration in OHCA patients was influenced by factors in addition to the duration of cardiac arrest, including vasopressors, high arterial oxygen partial pressure, and decreased renal function [14, 45]. Serum lactate seems to be a comprehensive marker of severity in OHCA patients [46], and the association of serum lactate concentrations and neurological outcome has been described in many previous studies [14–17, 47, 48]. In this study, both crude and predicted probability of 30-day favorable neurological outcomes decreased with increasing serum lactate concentration after ROSC (Fig. 2). Although serum lactate in OHCA patients does not necessarily reflect brain metabolites [49], it does reflect OHCA severity after ROSC.

The finding that TTM at 32–34 °C was not associated with predicted neurological outcomes in patients with mild or moderate hyperlactatemia was consistent with the results of the TTM trial, which did not show a significant benefit of TTM at 33 °C compared with 36 °C [4]. The lactate concentration of 6.7 ± 4.5 mmol/l in the TTM study population was thought to account for the lack of demonstrated benefit of TTM at 33 °C. The mean lactate concentration in both temperature groups evaluated in the TTM trial was similar to the concentration in the mild to moderate hyperlactatemia patients in our current study. Thus, post hoc analysis of the TTM trial results did not identify a patient subset that benefited from TTM at 33 °C compared with 36 °C [9, 10].

The present study had several limitations. First, this was a post hoc analysis. Although we calculated predicted probability using multiple logistic regression analysis, selection bias and uncontrolled confounding variables could have influenced the results. For example, the target temperature depended upon the physician’s choice, and more than 70% of patients received 32–34 °C (Table 2). The physicians had a tendency to assign patients with unstable hemodynamics to the 35–36 °C group to avoid hypotension and arrhythmia caused by 32–34 °C [50, 51]. However, there were no significant differences in crude survival and predicted probability of 30-day survival. Second, exclusion of the 3262 patients without TTM may affect the reliability of external validity. However, given that, of those, 1160 patients died at the emergency department, 89 patients reached Glasgow Coma Scale motor score of 6 after ROSC, and 2013 comatose admitted patients without TTM had much more severe prehospital characteristics than the patients finally analyzed (Table 1), it seemed reasonable that the physicians did not provide TTM to these patients. However, the responsibility for non-indication of TTM depended on each physician or each institution, so it could cause some selection biases. Third, our current study included only patients who completed TTM, which may have led to additional selection bias. However, 30-day survival rate in patients with TTM withdrawn was not one-third as high as that in those finally analyzed (22% vs 75%, *p* < 0.001, Additional file 2: Table S1) despite no significant difference in the proportion of TTM at 32–34 °C between two groups (73% vs. 79%, *p* = 0.198). Adverse events of TTM seemed unlikely to cause this profound difference. We thought that there were so many patients without indication of TTM among these patients. On the other hand, exclusion of the patient with missing lactate concentration may result in selection bias and decreased generalizability. In addition, we divided patients into 32–34 °C or 35–36 °C according to “targeted” temperature declared by each physician or each institute. We did not evaluate actual temperature and how strictly TTM was performed. Patient outcome might have been affected by the time needed to achieve target temperature during the induction phase, temperature fluctuation during the maintenance phase, and the rewarming rate [52]. Lastly, we could not assess long-term neurological outcomes.

## Conclusions

In OHCA patients with severe hyperlactatemia, the adjusted predicted probability of 30-day favorable neurological outcome was greater with 32–34 °C than with 35–36 °C. Further study is needed to determine whether 32–34 °C can improve neurological outcome in patients with severe hyperlactatemia.

## Supplementary information

**Additional file 1: Figure S1.** Serum lactate concentration and crude and adjusted predicted probability of favorable 30-day neurological outcomes.

**Additional file 2: Table S1.** Univariate analysis and multiple logistic regression models to obtain the adjusted predicted probabilities of 30-day favorable neurological outcome.

**Additional file 3: Table S2.** Univariate analysis and multiple logistic regression models to obtain the adjusted predicted probabilities of 30-day survival.

**Additional file 4: Figure S2.** Adjusted predicted probability of 30-day survival of 32–34 °C and 35–36 °C among patients in the three hyperlactatemia group.

## Data Availability

The data that support the findings of this study are available from the JAAM-OHCA registry committee but restrictions apply to the availability of these data, which were used under license for the current study, and so are not publicly available. Data are however available from the authors upon reasonable request and with permission of the JAAM-OHCA registry committee.

## Abbreviations

OHCA: out-of-hospital cardiac arrest
TTM: targeted temperature management
ROSC: return of spontaneous circulation
JAAM-OHCA registry: Japanese Association for Acute Medicine out-of-hospital cardiac arrest registry
CPR: cardiopulmonary resuscitation
AED: automated external defibrillator
CPC score: cerebral performance category score
ECMO: extracorporeal membrane oxygenation
IABP: intra-aortic balloon pumping
CI: confidence interval
