# Supplementary material for: Targeted temperature management guided by the severity of hyperlactatemia for out-of-hospital cardiac arrest patients: a post hoc analysis of a nationwide, multicenter prospective registry
Source: Ann Intensive Care. 2019 Nov 19;9:127. doi: 10.1186/s13613-019-0603-y (PMC6864017; doi:10.1186/s13613-019-0603-y)
Supplement: Supplementary file 1 — Additional file 1: Figure S1. Serum lactate concentration and crude and adjusted predicted probability of favorable 30-day neurological outcomes. [file 13613_2019_603_MOESM1_ESM.docx]

**Additional file 1**

**
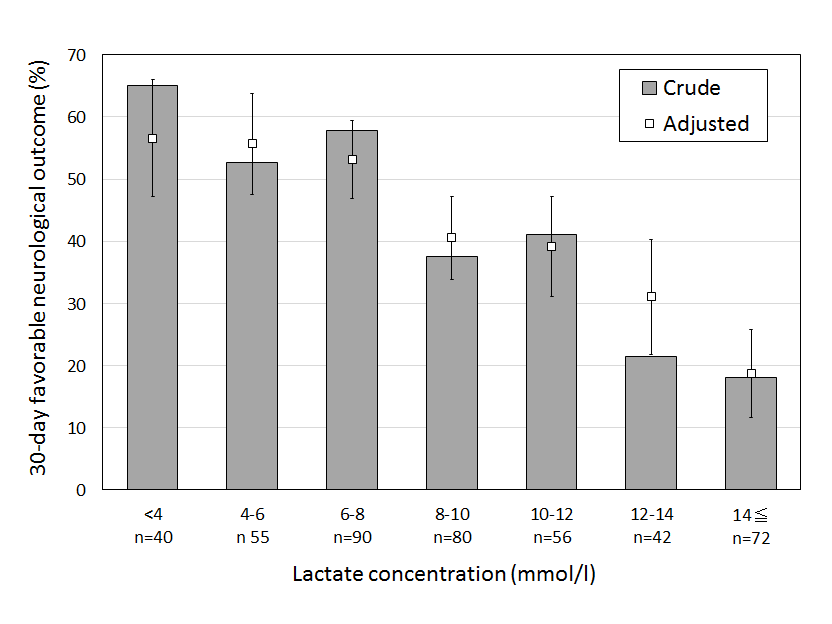
**

**Figure S1** Serum lactate concentration and crude and adjusted predicted probability of favorable 30-day neurological outcomes

Error bars indicate 95% confidence intervals.
