# Supplementary material for: Targeted temperature management guided by the severity of hyperlactatemia for out-of-hospital cardiac arrest patients: a post hoc analysis of a nationwide, multicenter prospective registry
Source: Ann Intensive Care. 2019 Nov 19;9:127. doi: 10.1186/s13613-019-0603-y (PMC6864017; doi:10.1186/s13613-019-0603-y)
Supplement: Supplementary file 2 — Additional file 2: Table S1. Univariate analysis and multiple logistic regression models to obtain the adjusted predicted probabilities of 30-day favorable neurological outcome. [file 13613_2019_603_MOESM2_ESM.docx]

**Additional file 2**

|  | Univariate analysis | | multiple logistic regression analysis ^a^ | |
| --- | --- | --- | --- | --- |
| Variables | OR (95%CI) | p value | Adjusted OR (95% CI) | p value |
| Age > 65 years | 0.47 (0.31-0.70) | < 0.01 | 0.38 (0.22-0.66) | < 0.01 |
| Male sex | 1.36 (0.86-2.17) | 0.19 | 0.83 (0.43-1.61) | 0.58 |
| Witness | 1.78 (1.08-2.95) | 0.02 | 1.95 (0.99-3.84) | 0.06 |
| Dispatcher instruction | 1.36 (0.93-2.00) | 0.11 | 1.11 (0.59-2.06) | 0.75 |
| Bystander-performed CPR | 2.04 (1.39-3.02) | < 0.01 | 1.53 (0.83-2.82) | 0.17 |
| Cardiac etiology | 3.95 (2.32-6.74) | < 0.01 | 1.76 (0.75-4.14) | 0.19 |
| Initial shockable rhythm | 4.02 (2.66-6.07) | < 0.01 | 2.68 (1.44-5.00) | < 0.01 |
| Prehospital epinephrine administration | 0.27 (0.16-0.46) | < 0.01 | 0.36 (0.18-0.73) | < 0.01 |
| Prehospital advanced airway management | 0.34 (0.22-0.53) | < 0.01 | 0.48 (0.27-0.86) | 0.01 |
| Time from call to hospital arrival, min | 0.96 (0.90-1.03) | 0.30 | 0.97 (0.95-1.00) | 0.04 |
| Prehospital ROSC | 9.81 (6.03-16.00) | < 0.01 | 10.30 (5.47-19.40) | < 0.01 |
| Glasgow Coma Scale score | 1.48 (1.25-1.74) | < 0.01 | 1.21 (0.99-1.46) | 0.06 |
| Coronary angiography | 2.70 (1.80-4.07) | < 0.01 | 1.48 (0.75-2.91) | 0.26 |
| ECMO and/or IABP | 0.81 (0.53-1.24) | 0.34 | 0.89 (0.47-1.69) | 0.72 |
| PaCO2 30-50 mm Hg | 3.93 (2.62-5.89) | < 0.01 | 2.12 (1.24-3.61) | < 0.01 |

**Table S1** Univariate analysis and multiple logistic regression models to obtain the adjusted predicted probabilities of 30-day favorable neurological outcome

*OR* odds ratio, *CI* confidence interval, *CPR* cardiopulmonary resuscitation, *ROSC* return of spontaneous circulation, *ECMO* extracorporeal membrane oxygenation, *IABP* intra-aortic balloon pumping,

^a^ The area under the receiver-operating-characteristic curve of the multiple logistic regression to calculate a predicted probability was 0.882.
