# Supplementary material for: Targeted temperature management guided by the severity of hyperlactatemia for out-of-hospital cardiac arrest patients: a post hoc analysis of a nationwide, multicenter prospective registry
Source: Ann Intensive Care. 2019 Nov 19;9:127. doi: 10.1186/s13613-019-0603-y (PMC6864017; doi:10.1186/s13613-019-0603-y)
Supplement: Supplementary file 3 — Additional file 3: Table S2. Univariate analysis and multiple logistic regression models to obtain the adjusted predicted probabilities of 30-day survival. [file 13613_2019_603_MOESM3_ESM.docx]

**Additional file 3**

|  | Univariate analysis | | multiple logistic regression analysis ^a^ | |
| --- | --- | --- | --- | --- |
| Variables | OR (95%CI) | p value | Adjusted OR (95% CI) | p value |
| Age > 65 years | 1.00 (0.65-1.55) | 1.00 | 0.99 (0.58-1.67) | 0.96 |
| Male sex | 1.03 (0.62-1.73) | 0.90 | 0.68 (0.36-1.26) | 0.22 |
| Witness | 1.65 (0.99-2.75) | 0.06 | 1.85 (1.00-3.43) | 0.05 |
| Dispatcher instruction | 1.74 (1.12-2.73) | 0.02 | 1.52 (0.84-2.78) | 0.17 |
| Bystander-performed CPR | 1.77 (1.14-2.74) | 0.01 | 1.18 (0.65-2.12) | 0.59 |
| Cardiac etiology | 1.87 (1.16-3.03) | 0.01 | 1.36 (0.68-2.72) | 0.39 |
| Initial shockable rhythm | 2.49 (1.60-3.89) | < 0.01 | 1.94 (1.07-3.50) | 0.03 |
| Prehospital epinephrine administration | 0.49 (0.31-0.80) | < 0.01 | 0.50 (0.27-0.91) | 0.02 |
| Prehospital advanced airway management | 0.84 (0.53-1.31) | 0.44 | 1.48 (0.85-2.60) | 0.17 |
| Time from call to hospital arrival, min | 0.99 (0.98-1.01) | 0.43 | 0.98 (0.96-1.01) | 0.20 |
| Prehospital ROSC | 5.75 (3.56-9.29) | < 0.01 | 4.47 (2.56-7.81) | < 0.01 |
| Glasgow Coma Scale score | 1.23 (1.01-1.51) | 0.04 | 1.03 (0.85-1.26) | 0.75 |
| Coronary angiography | 1.49 (0.96-2.30) | 0.07 | 1.21 (0.62-2.35) | 0.58 |
| ECMO and/or IABP | 0.57 (0.36-0.90) | 0.02 | 0.52 (0.28-0.99) | 0.05 |
| PaCO2 30-50 mm Hg | 2.38 (1.47-3.84) | < 0.01 | 1.46 (0.84-2.54) | 0.18 |

**Table S2** Univariate analysis and multiple logistic regression models to obtain the adjusted predicted probabilities of 30-day survival

*OR* odds ratio, *CI* confidence interval, *CPR* cardiopulmonary resuscitation, *ROSC* return of spontaneous circulation, *ECMO* extracorporeal membrane oxygenation, *IABP* intra-aortic balloon pumping,

^a^ The area under the receiver-operating-characteristic curve of the multiple logistic regression to calculate a predicted probability was 0.784.
