# Supplementary material for: Targeted temperature management guided by the severity of hyperlactatemia for out-of-hospital cardiac arrest patients: a post hoc analysis of a nationwide, multicenter prospective registry
Source: Ann Intensive Care. 2019 Nov 19;9:127. doi: 10.1186/s13613-019-0603-y (PMC6864017; doi:10.1186/s13613-019-0603-y)
Supplement: Supplementary file 4 — Additional file 4: Figure S2. Adjusted predicted probability of 30-day survival of 32–34 °C and 35–36 °C among patients in the three hyperlactatemia group. [file 13613_2019_603_MOESM4_ESM.docx]

**Additional file 4**

**
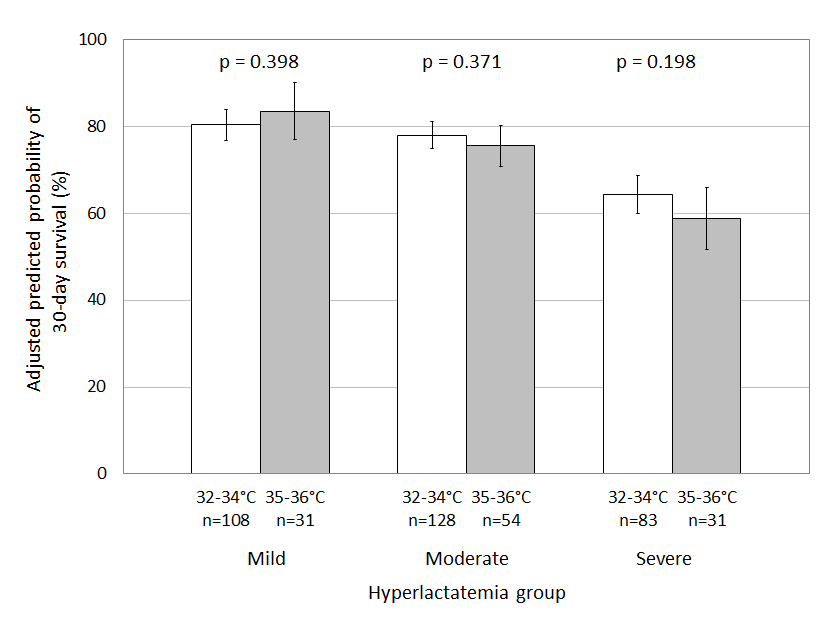
**

**Figure S2** Adjusted predicted probability of 30-day survival of 32-34 ºC and 35-36 ºC among patients in the three hyperlactatemia group

Error bars indicate 95% confidence intervals.

p for interaction = 0.087
